# A Case of Basal Ganglia Intraparenchymal Hemorrhage Following Lumbar Spinal Surgery

**DOI:** 10.7759/cureus.65692

**Published:** 2024-07-29

**Authors:** Michael J Gigliotti, Neel Patel, Chanju Fritch, Ephraim W Church, George T Reiter, Hajoe Park

**Affiliations:** 1 Neurosurgery, Penn State Health Milton S. Hershey Medical Center, Hershey, USA; 2 Neurosurgery, Penn State Health, Hershey, USA; 3 Neurosurgery, Penn State University College of Medicine, Milton S. Hershey Medical Center, Hershey, USA

**Keywords:** intracranial hemorrhage, neurosurgery, cerebrospinal fluid leak, spine, intraparenchymal hemorrhage

## Abstract

We report on a rare case of basal ganglia intraparenchymal hemorrhage with intraventricular extension occurring after a lumbar spinal surgery. A 65-year-old female presented for an elective L4-L5 lumbar laminectomy and posterior spinal fixation. Her initial operation was complicated by a cerebrospinal fluid (CSF) leak repaired with a dural synthetic graft. Her immediate post-operative course was complicated by delayed emergence, eye-opening apraxia, and left-sided hemiplegia and subsequent computed tomography (CT) of the head demonstrated a right-sided basal ganglia intraparenchymal hemorrhage (IPH) with intraventricular extension. CT angiogram of the head was unremarkable. She was taken back to the operating room for right-sided decompressive hemicraniectomy and external ventricular drainage (EVD) for hydrocephalus. Her EVD was discontinued on post-bleed day 13 and she was discharged on post-bleed day 14 to a long-term care facility with a modified Rankin scale (mRS) score of 6. She returned for a cranioplasty six months later, and on her last follow-up at nine months, had a mRS of 4 with persistent confusion and severe left-sided hemiparesis but was able to form simple sentences. In summary, intracranial hemorrhage is a rare complication of spine surgery, occurring in a small percentage of the population. Lobar IPH following spinal surgery is a rare complication, and has been hypothesized to be a result of excessive CSF loss during durotomy.

## Introduction

Intracranial hemorrhage (ICH) is a rare complication of spine surgery, with a proposed incidence rate of 0.8% [1]. Common causes of spontaneous ICH in the general population are hypertension, primary amyloid angiopathy (in patients older than 70 years of age), hemorrhagic infarction, septic embolism, mycotic aneurysm, humoral hemorrhage, coagulopathy, anticoagulant drug use, infection, and trauma [1]. The etiology of intracranial hemorrhage as a complication of spinal surgery is unclear, but it has been postulated that it may be caused by excessive cerebrospinal fluid (CSF) loss caused by dural tearing [1]. Hemorrhages reported have included primarily in the cerebellum, but supratentorial hemorrhages occur to a lesser extent. In a literature review by Al-Saadi et al. [2] reviewing 79 studies of post-operative ICH following spinal surgery identified 109 patients with post-operative ICH. Of these 109 cases, cerebellar hemorrhages occurred in 56.3% of patients with intraparenchymal hemorrhages (IPH) occurring in 17.9% of cases. Other intracranial bleeds included subdural hematoma (SDH), subarachnoid hemorrhage (SAH), and epidural hematoma (EDH) [2]. We report a rare case of basal ganglia intraparenchymal hemorrhage with intraventricular extension occurring after a lumbar spinal surgery.

## Case presentation

**Figure 1:**
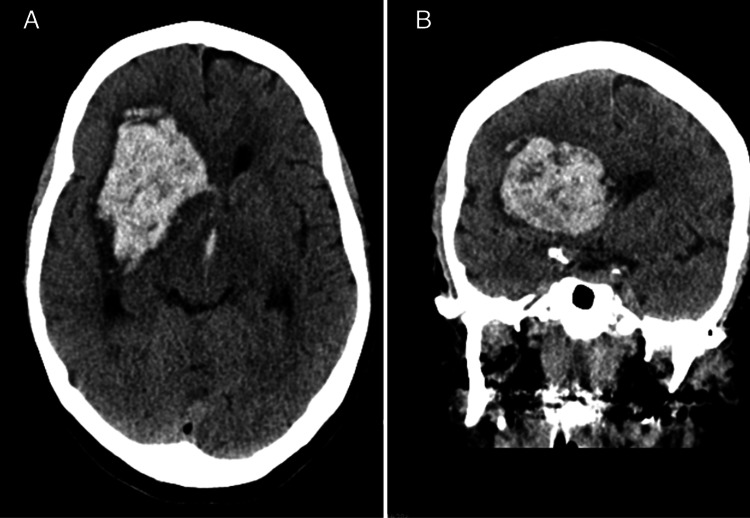
Axial (A) and coronal (B) CT of the head showing right-sided basal ganglia intraparenchymal hemorrhage measuring 8.5 × 4.3 × 3.8 cm with associated brain compression, midline shift, intraventricular extension, and mild perilesional edema.

The patient is a 65-year-old female with a past medical history of hyperlipidemia, mitral valve prolapse, and anxiety who initially presented for an elective L4-L5 lumbar laminectomy and fusion lasting approximately 3 hours. Systolic blood pressure was under 140 mmHg during induction and ranged from 80 mmHg to 120 mmHg through the duration of the case. Her initial operation was complicated by a small dural tear that resulted in a cerebrospinal fluid (CSF) leak that was repaired with Duragen at the time of the operation. Her immediate post-operative course was complicated by delayed emergence from anesthesia followed by eye-opening apraxia, and left-sided hemiplegia approximately 2 hours following the conclusion of her surgery, at which time she was taken for an emergent computed tomography (CT) scan of her head without contrast. Her systolic blood pressure ranged from 69 mmHg to 124 mmHg and she was never hypertensive post-operatively prior to her first imaging study. Her CT scan demonstrated a right-sided basal ganglia intraparenchymal hemorrhage with intraventricular extension measuring 8.5 × 4.3 × 3.8 cm (Figure 1) and CT angiogram of the head was unremarkable for vascular lesions.

**Figure 2:**
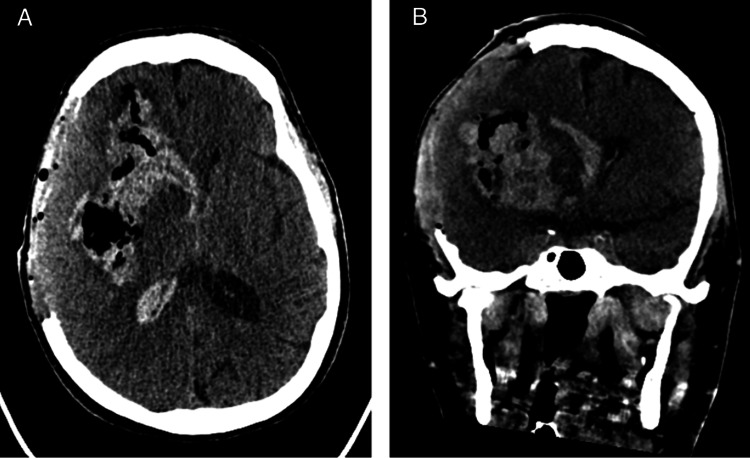
Axial (A) and coronal (B) post-operative CT of the head following right-sided decompressive hemicraniectomy.

She was taken emergently to the operating room for a right-sided frontotemporal decompressive craniectomy with the evacuation of her hematoma. Post-operative imaging demonstrated a good bony decompression of greater than 12 cm but persistent intraparenchymal and intraventricular blood (Figure 2).

An external ventricular drain was placed after her post-operative CT given persistent ventricular blood. Her hospital course was complicated by electrographic seizures requiring Keppra as well as acute hypoxic respiratory failure and dysphagia necessitating long-term ventilator dependence. She was subsequently taken back to the operating room for tracheostomy placement and percutaneous gastrostomy (PEG) placement on post-bleed day 9. The rest of her hospital course was uncomplicated and her external ventricular drain was discontinued on post-bleed day 13 and she was discharged on post-bleed day 14 to a long-term assisted care facility.

**Figure 3:**
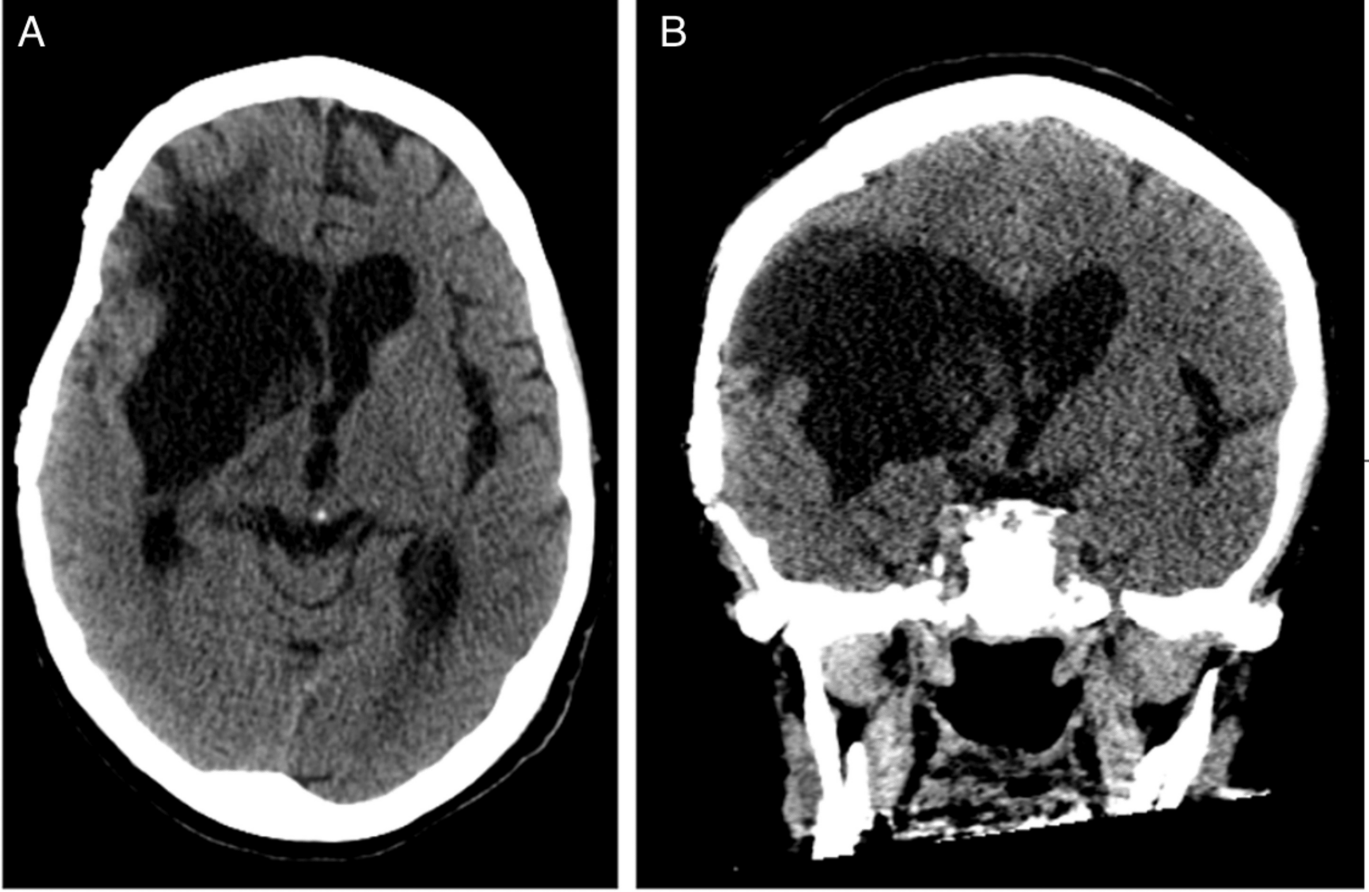
CT of the head performed 9 months following intraparenchymal hemorrhage. Axial (A) and coronal (B) images show encephalomalacia in the prior intraparenchymal hemorrhage bed with resolution of her midline shift following cranioplasty.

She returned for an autologous cranioplasty six months later, and on her last follow-up at nine months she had since had her tracheostomy tube and PEG tube removed. She was able to form simple sentences but remained confused with significant left-sided hemiparesis (mRS score of 4). She had an interval CT of the head, which showed right-sided basal ganglia encephalomalacia (Figure 3).

## Discussion

A rare phenomenon, intracranial hemorrhage (ICH) following spinal surgery may lead to persistent neurological impairment and death. Presenting symptoms are nonspecific and ICH typically occurs in females more than males [2]. In this case, we describe a female with unilateral ICH and intraventricular extension following a L4-L5 laminectomy and posterior spinal fusion that was complicated by a dural tear intraoperatively resulting in a minor cerebrospinal fluid (CSF) leak and repaired with a dural synthetic graft.

Several risk factors have been associated with spontaneous intracranial hemorrhage, including hypertension, amyloid angiography, vascular malformations, anticoagulant use, tumors, trauma, and infection [3]. However, the etiology of ICH following spinal surgery is less clear. In one review, dural tears were reported in 77.1% of cases of patients found to have ICH as a complication of spinal surgery and thus have been proposed as an inciting event leading to spontaneous intracranial bleeds [2]. Dural tears resulting in excessive CSF loss, however, typically result in hemorrhages that are venous in etiology (i.e., cerebellar hemorrhages) via downward cerebellar displacement caused by intracranial hypotension [4]. The aforementioned proposed hypothesis in this case, however, is less likely given the presumed arterial source of the patient's ICH. Intraoperative positioning also may play a role, with reports of patients in sitting or lateral positions suffering from postoperative ICH, however, no link has been established [4-6]. In addition, no correlation has been noted between post-operative ICH and age, sex, type of intervention, or pathology operated on [4].

Intracranial hemorrhage following spine surgery typically is found in the cerebellum and reported cases of supratentorial ICH are very rare. A case report by Berry et al. [7], purported to be the first case report of supratentorial basal ganglia hemorrhage following a cervical fusion, has a bilateral distribution with intraventricular extension. Interestingly, our case involves a case of unilateral bleeding. This likely dispels the notion that venous hemorrhage caused ICH in this case as a result of intracranial hypotension and lends more credence to the fact that our patient's bleed arose from an arterial source. In light of this, attributing an etiology to the patient's bleed from excessive CSF loss from an intraoperative dural tear is unlikely. Instead, the appearance of perilesional edema on her initial CT following her surgery may suggest that she already had a developing (or existing) spontaneous intraparenchymal bleed prior to (or at the time of) her original surgery. Hypertension during induction of anesthesia is not uncommon [8], and thus could be a contributing factor in the formation of a spontaneous ICH. The patient presented here did not have any documented episodes of hypertension in the preoperative period and was at her baseline neurological examination before her original surgery as well. However, presenting symptoms of ICH may be nonspecific and thus may have been overlooked prior to her surgery, and time to diagnosis was delayed given her prolonged exposure to anesthesia and delayed emergence from anesthetic as well. Conversely, the patient was hypotensive in the post-operative period, thus hypotension could have led to cerebral hypoperfusion resulting in an ischemic stroke and early hemorrhagic conversion. Other etiologies of spontaneous hemorrhages, such as from iatrogenic coagulopathy or cerebral amyloid angiopathy, are less likely given that the patient was not administered any blood thinning agents or anticoagulants in the preoperative period and given her age, respectively.

## Conclusions

We report a care case of unilateral basal ganglia intracranial hemorrhage with intraventricular extension of unknown etiology. Patients with delayed ICH following spinal surgery are likely an underrepresented cohort given the lack of routine post-operative intracranial imaging studies performed post-operatively in the absence of lateralizing signs on examination and nonspecific symptoms, such as headaches. Surgeons should be aware of the potential morbidity and mortality of post-operative ICH, and consideration should be taken in the pre-operative evaluation and consent to this potential complication. Management is based on the severity of findings on the patient’s neurological examination as well as the size and associated brain compression of the bleed. Emergency surgery may be warranted in select patients, however, higher ICH scores tend to lead to a higher mortality. In the post-operative period, early detection and intervention is essential to ward off the most devastating effects of this complication.
